# Sirt1 inhibits macrophage polarization and inflammation in gouty arthritis by inhibiting the MAPK/NF-κB/AP-1 pathway and activating the Nrf2/HO-1 pathway

**DOI:** 10.1007/s00011-024-01890-9

**Published:** 2024-05-13

**Authors:** Xu Zhao, Menglan Li, Yiwei Lu, Mi Wang, Jiawei Xiao, Qingqing Xie, Xinyi He, Shiquan Shuai

**Affiliations:** 1 Department of Rheumatology and Immunology, Nanchong Central Hospital (Nanchong Clinical Research Center), The Second Clinical Medical College of North Sichuan Medical College, No. 97, Nanlu, Shunqing District, Nanchong, 637000 Sichuan China; 2 Nanchong Key Laboratory of Inflammation and Immunization, Nanchong Central Hospital (Nanchong Clinical Research Center), The Second Clinical Medical College of North Sichuan Medical College, Nanchong, 637000 Sichuan China

**Keywords:** Sirt1, MAPK signaling pathway, Oxidative stress, Macrophage polarization, Gouty arthritis

## Abstract

**Objective and design:**

To elucidate Sirt1’s role in gouty arthritis inflammation and its potential mechanisms.

**Material:**

Constructed murine models of gouty arthritis and conducted THP-1 cell experiments.

**Treatment:**

1 mg of MSU crystals injected into mice ankle joints for a 72-h intervention. After a 3-h pre-treatment with Sirt1-specific inhibitor (EX527) and agonist (SRT2104), inflammation was induced for 21 h using lipopolysaccharide (LPS) plus MSU crystals.

**Methods:**

We assessed gouty arthritis severity through joint inflammation index, swelling, and hematoxylin and eosin (H&E) staining, and measured CD68 mononuclear macrophages and Sirt1 expression in synovial tissue via immunohistochemistry. ELISA, NO assay, RT-qPCR, Flow cytometry, and Western blot were utilized to examine macrophage inflammatory factors, polarization, reactive oxygen species(ROS), MAPK/NF-κB/AP-1 and Nrf2/HO-1 pathways proteins.

**Results:**

Significant joint swelling, synovial tissue edema, and inflammatory cell infiltration were observed. CD68 mononuclear macrophages and Sirt1 expression were elevated in synovium. Sirt1 activation decreased inflammatory factors, M1 polarization, and ROS generation. Sirt1 activation reduced p38/JNK phosphorylation, thereby inhibiting downstream NF-κB p65/AP-1 and enhancing Nrf2/HO-1, thus suppressing inflammation.

**Conclusions:**

Sirt1 alleviates M1 macrophage polarization and inflammation in gouty arthritis by inhibiting the MAPK/NF-κB/AP-1 pathway and activating the Nrf2/HO-1 pathway. Thus, activating Sirt1 may provide a new therapeutic target for gouty arthritis.

## Introduction

Gouty arthritis is a local or systemic inflammatory disease due to the deposition of monosodium urate (MSU) crystals in the joints and(or) surrounding tissues. It is characterized by high prevalence, frequent relapses, significant disability, and lifelong impacts. It is clinically manifested as local redness, swelling, heat, and pain of the joint, accompanied by mobility limitation. If not treated timely, it can recur and progress to chronic gouty arthritis. This progression can give rise to the formation of tophi and even have detrimental effects on the kidneys, resulting in gouty nephropathy and renal failure. Such consequences have a profound impact on the quality of life of patients and impose substantial economic burdens on both families and society[1].

Macrophage polarization is closely associated with inflammation in gouty arthritis. MSU crystals can change macrophage M1/2 polarization by transmitting extracellular signals to macrophages through various signaling pathways. This, in turn, mediates the inflammatory response by releasing pro-inflammatory cytokines, chemokines, and other inflammatory factors either alone or in combination [2–6]. Among these, the mitogen-activated protein kinase (MAPK) signaling pathway is known for promoting the inflammatory cascade. The MAPK signaling pathway mainly consists of three components, namely extracellular signal-regulated kinases 1/2 (ERK1/2), c-Jun N-terminal kinase (JNK), and P38 MAPK. It primarily activates nuclear factor-kappa B (NF-κB) and activator protein-1 (AP-1) through phosphorylation of these three proteins, thereby regulating the inflammatory response [7]. Furthermore, heme oxygenase-1 (HO-1), strictly regulated by MAPK-mediated nuclear factor erythroid 2-related factor 2 (Nrf2) activation, is crucial in inhibiting the production of reactive oxygen species (ROS) and pro-inflammatory cytokines in pro-inflammatory macrophages [8, 9]. However, the current study on how to alter macrophage polarization via the MAPK signaling pathway is relatively limited.

Sirt1 is a nicotinamide adenine dinucleotide dependent class III histone deacetylase, which can regulate the activity of various transcription factors, such as p53, NF-κB, Nrf2, HIF1α, Notch, Forkhead Box O proteins (FoxOs), peroxisome proliferator-activated receptor gamma coactivator 1-Alpha (PGC-1α), and peroxisome proliferator-activated receptor gamma (PPARγ) through deacetylation [10]. Through these actions, Sirt1 could regulate various physiological functions, like cell differentiation [11], apoptosis [12], autophagy [13], metabolism [14], inflammation [15], and oxidative stress [16]. Studies have confirmed that Sirt1 can modulate macrophage M1/M2 polarization and suppress arthritis inflammation by inactivating the MAPK/NF-κB signaling pathway [17]. Moreover, Sirt1 can regulate macrophage-mediated inflammation by inhibiting the transcriptional activity of AP-1 and the expression of downstream gene COX-2 [18]. However, it is still not fully understood whether Sirt1 alleviates inflammation in gouty arthritis by regulating macrophage polarization.

In this study, we confirmed the expression of CD68 mononuclear macrophages and Sirt1 in the synovium of mice with MSU crystals-induced gouty arthritis. Furthermore, THP-1 cells were induced with LPS + MSU crystals to explore whether activation or inhibition of Sirt1 affects macrophage polarization and inflammatory responses through the MAPK and Nrf2/HO-1 pathways. The potential mechanisms were also investigated.

## Materials and methods

### Animal experiment

#### MSU crystal preparation

MSU crystals were prepared according to previously reported methods with some modifications [19]. Uric acid (1 g, U0881-10G, Sigma-Aldrich) was dissolved in 200 mL of boiling water, and the pH was adjusted to 8.9 by adding 1 M NaOH. The mixture was allowed to crystallize overnight at room temperature. The precipitate was filtered out of the solution and dried at 42 °C. The dried crystals were weighed under sterile conditions and stored at room temperature. Prior to experiments, the crystals were diluted to the desired concentration using phosphate-buffered saline (PBS) and sterilized under high temperature and pressure.

#### Establishment of a murine model of gouty arthritis

Wild-type C57BL/6 mice (male, aged 6–8 weeks, weighing 20 ± 0.53 g) were purchased from Beijing Sipeifu Biotechnology Co., Ltd. To acclimate to the environment, all mice were housed under specific pathogen-free (SPF) conditions with a 12-h light/12-h dark cycle at 12–22 °C for 7 days, with free access to food and water during the experimental period. The mice were randomized into a blank control group and a model group, with 6 mice in each group, then weighed, and labeled accordingly. Prior to modeling, mice in both groups were anesthetized using 3% isoflurane inhalation, and the thickness of paw pads on both the left and right sides of each mouse was measured using a vernier caliper and recorded. The thickness of the paw pad was consistent among the mice with no significant difference. In the control group, 50 µL of sterile PBS was injected into the right posterior ankle joint of the mice. In the model group, 50 µL of sterile MSU crystal suspension (containing 1 mg of MSU crystals) was injected into the right posterior ankle joint of the mice. The swelling of the contralateral joint capsule indicated successful modeling of gouty arthritis in mice. All animal experiments were conducted following the Guide for the Care and Use of Laboratory Animals and approved by the Ethics Committee of North Sichuan Medical College (Ethical approval number: NSMC Ethics Animal Review [2023]099).

#### Joint inflammation index and swelling assessment

Based on the Coderre method [20], inflammation was assessed at 3, 6, 12, 18, 24, 48, and 72 h post-modeling and classified into four levels. Level 0 indicated normal toes with no signs of inflammation; Level 1 indicated mild swelling, redness on joint skin, clear bony landmarks, and mild inflammation; Level 2 indicates localized swelling and redness of the joint, with no visible bony landmarks and moderate inflammation; Level 3 indicated swelling beyond the joint area, severe inflammation, reduced weight-bearing capacity of the foot, and frequent lifting of the injured limb. The swelling was assessed according to the Zhang’s method [21]. The initial circumference of the right posterior ankle joint was measured using a thread before modeling (averaged) and marked. After successful modeling, the circumference of the right ankle joint was measured again at the marked location at 3, 6, 12, 18, 24, 48, and 72 h post-modeling (averaged), and joint swelling was calculated using the formula: joint swelling = circumference measured at each time point − initial circumference of the joint.

#### Hematoxylin and eosin (H&E) staining

Mice were euthanized 72 h after injection of MSU crystals, and ankle joint tissues were fixed in 4% paraformaldehyde for more than 24 h. The tissues were then removed and soaked in a pre-prepared 15% EDTA decalcification solution for decalcification, followed by dehydration, embedding, sectioning, dewaxing, H&E staining, and slide mounting. Finally, the tissue sections were photographed and examined under the microscope.The severity of the lesion follows the previous standards [22].

#### Immunohistochemistry

After paraffin-embedding, deparaffinization, antigen retrieval, and blockade of endogenous peroxidase activity, the sections were incubated for 20 min with bovine serum albumin (BSA) blocking solution, followed by overnight incubation with primary antibodies against CD68 (GB113109, Servicebio, 1:100) and Sirt1 (bs-0921R, Bioss, 1:50) at 4 °C. Afterward, tissue sections were incubated with secondary antibodies against Goat Anti-Rabbit IgG (H + L) HRP (GB23303, Servicebio, 1:100) at 37 °C for 30 min, followed by color development using 3,3′-Diaminobenzidine (DAB). Finally, the sections were counterstained with hematoxylin, dehydrated, and mounted, and images were captured for analyses.

### Cellular experiments

#### Materials and reagents

The materials and reagents used in this study mainly included Sirt1 specific inhibitor (EX527, purity: 99.89%, HY-15452, MCE), Sirt1 specific agonist (SRT2104, purity: 99.10%, HY-15262, MCE), lipopolysaccharide (LPS, L6386, Sigma-Aldrich), Phorbol 12-myristate 13-acetate (PMA, HY-18739, MCE), RPMI-1640 medium (PM150110, Procell), fetal bovine serum (FBS, C04001-500, Vivacell), and 1%penicillin streptomycin (P/S, S110JV, Shanghai BasalMedia Technologies Co., Ltd).

#### Cell culture and stimulation

THP-1 cells purchased from Shanen Biotechnology Co., Ltd. (Wuhan, China) had been fully certified under the Quality Management System (ISO9001:2015). The cells were cultured in RPMI-1640 medium supplemented with 10% FBS and 1% P/S at 37 °C with 5% (v/v) CO_2_. THP-1 cells were seeded in six-well plates at a density of 1 × 10^6^ cells/mL and divided into blank group, model group (LPS + MSU), EX527 group (EX527 + LPS + MSU), and SRT2104 group (SRT2104 + LPS + MSU). THP-1 cells were induced to differentiate into macrophages by incubation with 100 nM PMA for 48 h. The cells in the EX527 group and SRT2104 group were pretreated with 10 μM EX527 and 10 μM SRT2104, respectively, for 3 h. Subsequently, all groups of cells (except the blank group) were stimulated with LPS (1 μg/mL) for 3 h, followed by stimulation with MSU (200 μg/mL) for 18 h in the cell culture medium. The blank group received no treatment.

#### ELISA and NO assays

Cell culture supernatant was collected to detect the levels of IL-1β (ZC-32420, ZCIBIO), IL-6 (ZC-32446, ZCIBIO), PGE2 (ZC-34407, ZCIBIO), and TNF-α (ZC-35733, ZCIBIO) according to the manufacturer's instructions of the ELISA kits. Briefly, the ELISA kits were equilibrated at room temperature for 30 min. Then, standards and test samples (50 μL) of different concentrations were added to each standard well and sample well. Next, 100 μL of horseradish peroxidase (HRP)-conjugated detection antibody were added to each well of the standard and sample wells and incubated at 37 °C for 60 min in a constant temperature incubator. After the plate was washed 5 times, 50 μL of substrate A and B were added to each well and incubated at 37 °C in the dark for 15 min. Finally, 50 μL of stop solution was added to each well and the OD value of each well was measured at a wavelength of 450 nm within 10 min. The level of NO (A012-1-1, Nanjing Jiancheng Bioengineering Institute) in the cell culture supernatant was detected according to the manufacturer's instructions of the NO assay kit, and the OD value of each well was measured at a wavelength of 550 nm. The concentrations of cytokines were calculated based on the standard curve drawn from standard concentrations.

#### Real-time quantitative PCR

RT-qPCR was performed to detect the expression of COX-2, iNOS, and MCP-1 in THP-1 cells. According to the manufacturer's instructions, total RNA was extracted from the cells using a Trizol reagent and then reverse transcribed into cDNA using the HiScript^®^ III RT kit (#R323-01, Vazyme Biotech Co., Ltd., Nanjing, China). The primers for the genes were designed using NCBI/Primer Premier primer design software and synthesized by Shanghai Sangon Biotch Co., Ltd, followed by purification using ULTRAPAGE. Then, the cDNA was amplified by real-time PCR under the following conditions: initial denaturation at 95 °C for 30 s, followed by 45 cycles of denaturation at 95 °C for 5 s, annealing at 55 °C for 30 s, and extension at 72 °C for 30 s. With GAPDH as an internal reference, the relative expression levels were calculated using the 2 − ΔΔCt method. The primer sequences are Table 1.

**Table 1:** Primer sequences for RT-qPCR

Primer	Forward (5′ → 3′)	Reverse (5′ → 3′)
GAPDH	TGACTTCAACAGCGACACCCA	CACCCTGTTGCTGTAGCCAAA
COX-2	TGTCAAAACCGAGGTGTATGTA	AACGTTCCAAAATCCCTTGAAG
iNOS	GACTTTCCAAGACACACTTCAC	TTCGATAGCTTGAGGTAGAAGC
MCP-1	ACCAGCAGCAAGTGTCCCAAAG	TTTGCTTGTCCAGGTGGTCCATG

#### Flow cytometry

##### Macrophage polarization

After the cell pellet was collected and resuspended, FITC anti-human CD11b (301329, Biolegend) and APC anti-human CD86 (305411, Biolegend) were added for 30 min of incubation at 4 °C in the dark. Subsequently, 500 μL of Fixation Buffer was added for 20 min of incubation at room temperature. After centrifugation, the pellet was washed, resuspended, and then incubated with Intracellular Staining Perm Wash Buffer. Following another round of centrifugation, the cells were resuspended again and added with PE anti-human CD206 (321105, Biolegend) for 30 min of incubation at 4 °C. After centrifugation, the cells were washed with Intracellular Staining Perm Wash Buffer, centrifuged again, and resuspended. Finally, the cells were analyzed using flow cytometry.

##### ROS

The probes were loaded into the cells using 2,7-dichlorodihydrofluorescein diacetate (DCFH-DA). In brief, the cell pellet collected after intervention was treated with DCFH-DA (10 μM) and incubated at 37 °C in a cell culture incubator for 20 min. After washing and centrifugation, the supernatant was discarded, and the cells were resuspended in PBS. For the positive control group for ROS (Rosup), after loading the DCFH-DA probe, 1 mL of Rosup dilution (1:1000) was added and incubated at 37 °C in an incubator for 30 min. After washing and centrifugation, the supernatant was discarded, and the cells were resuspended in PBS. Finally, the cells were obtained for analysis using flow cytometry.

#### Western blot analysis

Total protein was extracted from the cells using RIPA lysis buffer, and the protein concentration was determined using the BCA assay kit. After denaturation at 95 °C, the protein was stored at − 80 °C. Equal amounts of protein were loaded to each lane and separated by 10% SDS-PAGE. The separated proteins were then transferred onto PVDF membranes and blocked with 5% skim milk powder for 2 h. The PVDF membranes were then incubated overnight at 4 °C with appropriate primary antibodies against β-actin (AC026, Abclonal, 1:50,000), Sirt1 (60303-1-lg, Proteintech, 1:2000), AP-1 (66313-1-Ig, Proteintech, 1:2000), NF-κB p65 (bs-0465R, Bioss, 1:2000), NF-κB p-p65 (82335-1-RR, Proteintech, 1:2000), p-P38 (bs-5476R, Bioss, 1:2000), P38 (bs-0637R, Bioss, 1:2000), p-JNK (80024-1-RR, Proteintech, 1:2000), JNK (66210-1-lg, Proteintech, 1:2000), HO-1 (ab85309, Abcam, 1:3000), and Nrf2 (16396-1-AP, Proteintech, 1:2000). After washing with TBST, the membranes were incubated at room temperature for 2 h with Goat Anti-Rabbit IgG (H + L) HRP (S0001, Affbiotech, 1:5000), followed by detection using enhanced chemiluminescence (ECL). The bands were exposed using the fluorescence imaging system V2.0 (Tanon, Shanghai, China), scanned, and analyzed using Gel-Pro analyzer 4.0. The relative expression levels of the target proteins were calculated with β-actin as an internal reference.

### Statistical analysis

The data were expressed as mean ± standard deviation (‾X ± SD). Differences between two groups were analyzed using independent samples t-test, while differences among multiple groups were analyzed using one-way ANOVA, followed by post-hoc LSD test. The analysis was conducted using SPSS 25.0 software (IBM, Armonk, NY, USA). P value < 0.05 was considered statistically significant.

## Results

### Joint inflammation index and swelling assessment

**Fig. 1:**
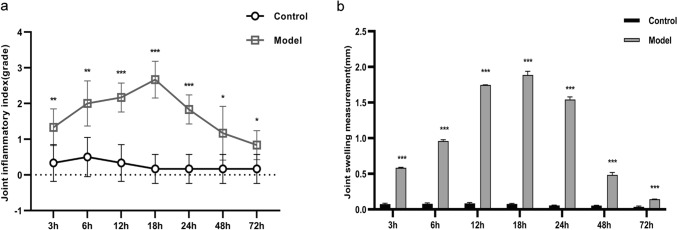
Effects of injecting 50 µL of sterile PBS and 50 µL of sterile MSU crystal suspension (1 mg) into the mouse ankle joint on joint inflammation. **a** Grading of joint inflammation index at different time points. **b** Measurement of joint swelling at different time points. Data were shown as mean ± SD (n = 6), *P < 0.05, **P < 0.01, ***P < 0.001 vs Control group

Compared to the control group, the ankle joint inflammation index and joint swelling degree significantly in the model group increased and peaked at around 18 h post-modeling (Fig. 1).

### Effects of MSU-induced gouty arthritis on the synovium and synovial inflammatory cell infiltration in mice

**Fig. 2:**
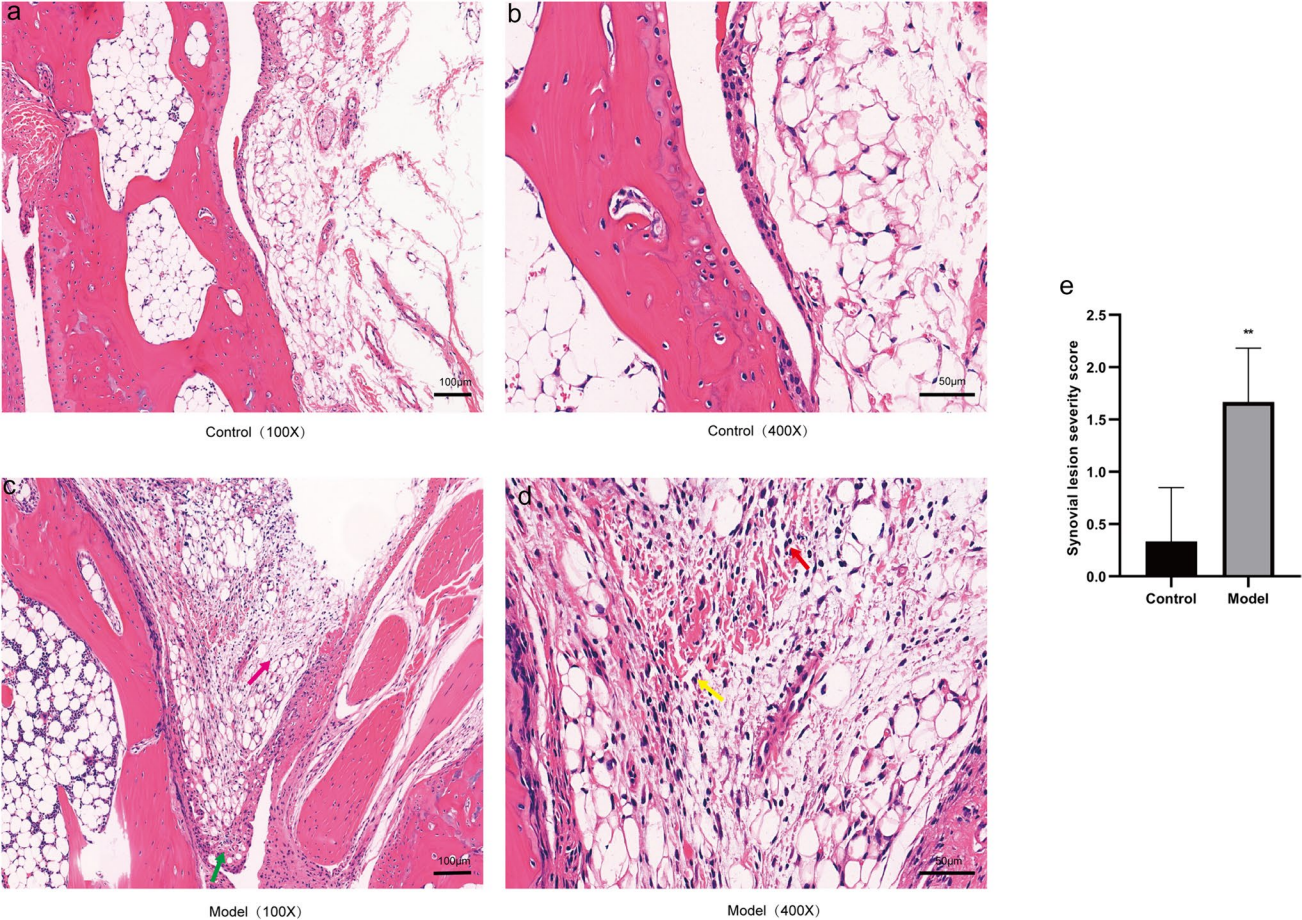
Effects of MSU-induced gouty arthritis on the synovium and synovial inflammatory cell infiltration in mice. **a, b** Synovial tissues of the control group at × 100 and × 400 magnifications. **c, d** Synovial tissues of the model group at × 100 and × 400 magnifications, with mononuclear cells indicated as red arrows, neutrophils as yellow arrows, synovial tissue edema as pink arrows, and synovial cell proliferation as green arrows. **e** Synovial lesion severity score. Data were expressed as mean ± SD (n = 6), *P < 0.05,**P < 0.01,***P < 0.001 vs Control group

Compared to the control group, the model group of mice exhibited synovial tissue edema, synovial cell proliferation, neovascularization, and infiltration of inflammatory cells (predominantly lymphocytes and neutrophils) (Fig. 2).

### Synovial CD68(+) mononuclear macrophages and Sirt1 expression in mice of MSU-induced gouty arthritis

**Fig. 3:**
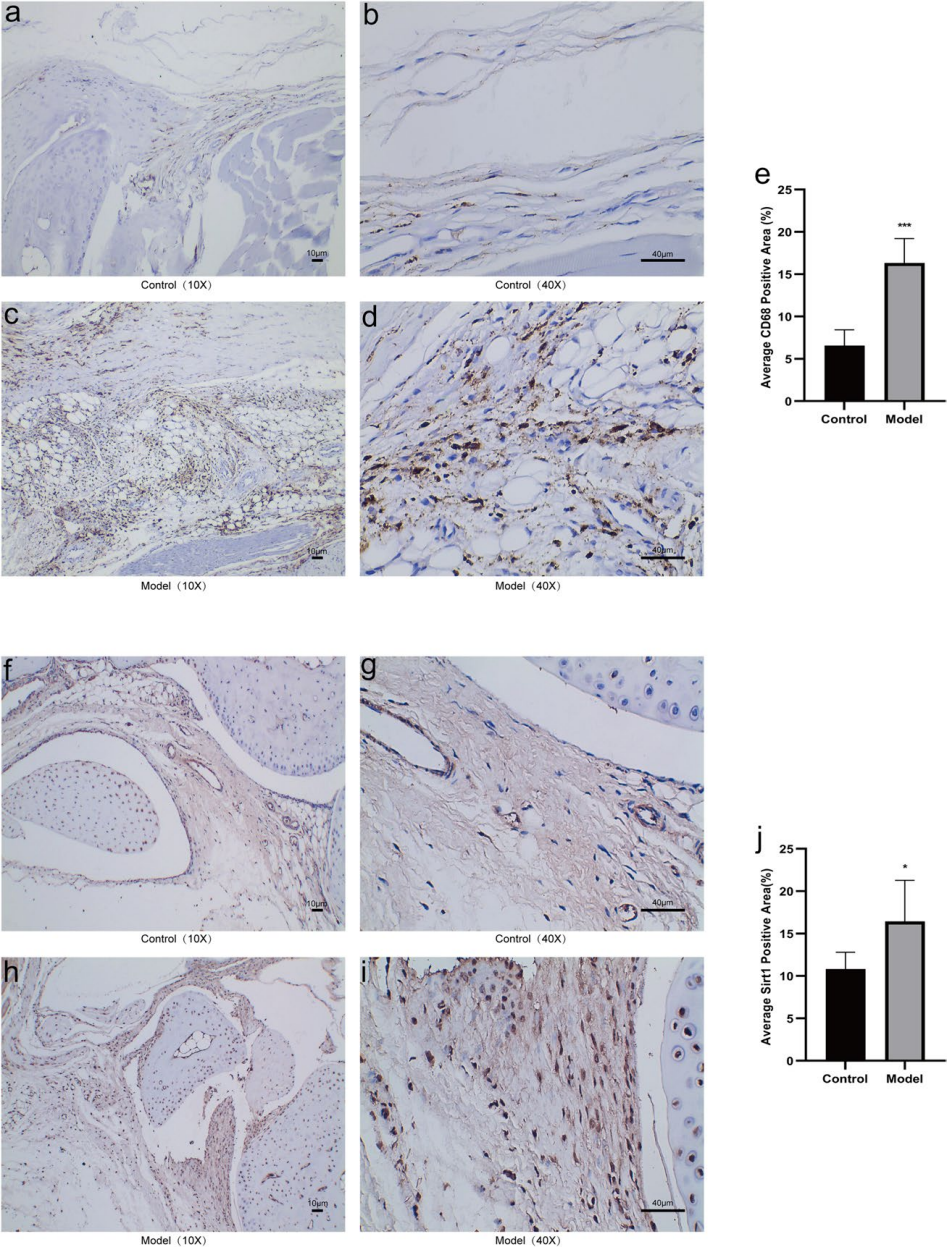
CD68 mononuclear macrophages and Sirt1 expression in synovial tissue of MSU-induced gouty arthritis were significantly increased. Following the injection of 50 µL of sterile PBS and 50 µL of sterile MSU crystal suspension (1 mg) into the right ankle joint of mice, the expression of CD68(+) mononuclear macrophages and Sirt1 in the synovium was assessed. **a–d** The expression of CD68(+) mononuclear macrophages in both the control group and the model group was assessed at both × 10 and × 40 magnifications. **f–i** The expression of Sirt1 in both the control group and the model group was assessed at both × 10 and × 40 magnifications. **e**, **j** Average CD68 and Sirt1 positive area. Data were expressed as mean ± SD (n = 6), *P < 0.05, **P < 0.01, ***P < 0.001 vs Control group

Compared to the control group, the model group of mice exhibited significant increases in CD68 mononuclear macrophages (P < 0.001) and Sirt1 protein expression in the synovial tissue of the ankle joint (P < 0.05) (Fig. 3). These results implied the involvement of mononuclear macrophages and Sirt1 in the development of gouty arthritis.

### Effect of activating and inhibiting Sirt1 on the levels of IL-1β, IL-6, TNF-α, PGE2, and NO in THP-1 Cells

**Fig. 4:**
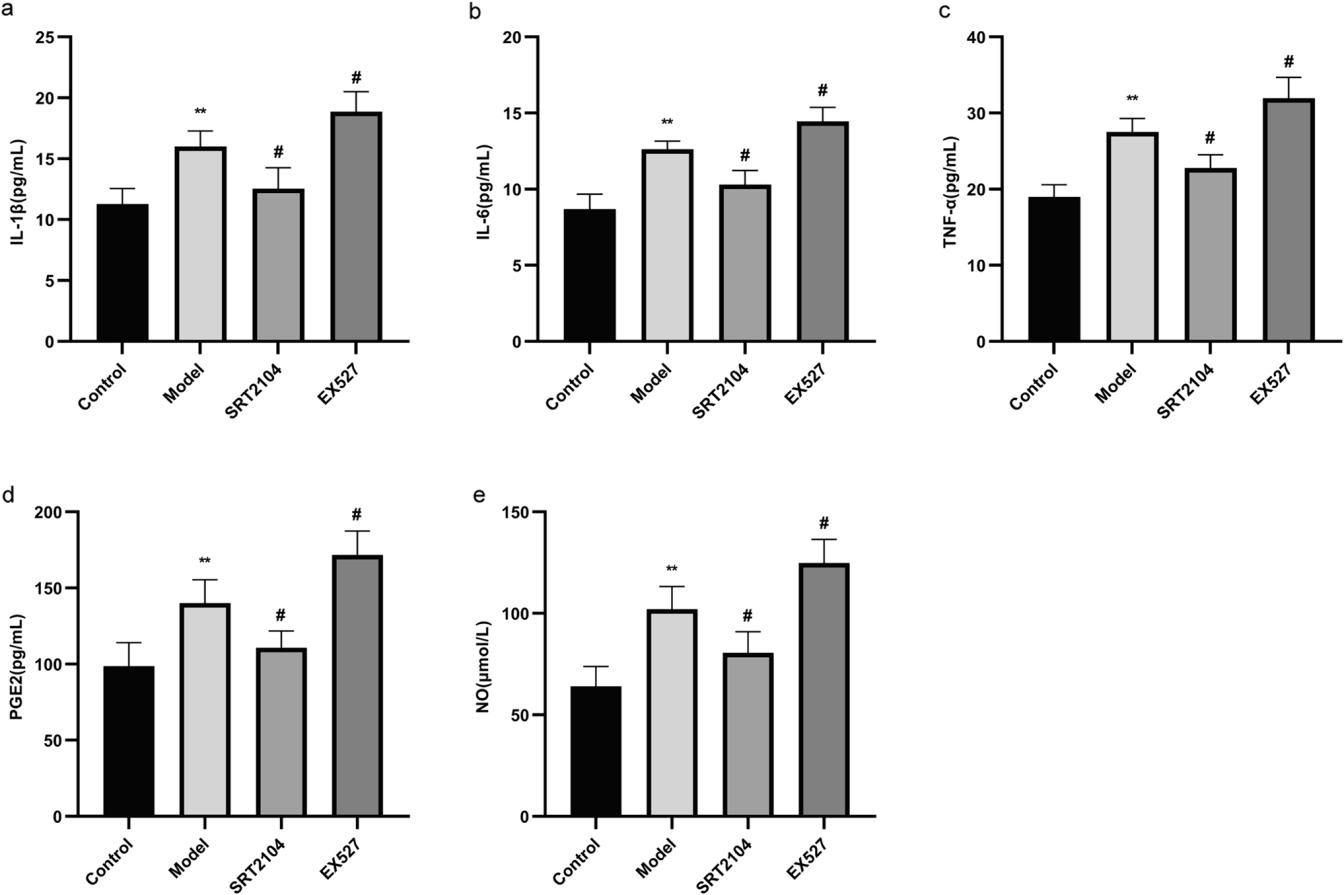
Effects of activating and inhibiting Sirt1 on the levels of IL-1β (**a**), IL-6 (**b**), TNF-α (**c**), PGE2 (**d**), and NO (**e**) in LPS plus MSU-activated THP-1 cells. Data were shown as mean ± SD (n = 3); *P < 0.05, **P < 0.01, ***P < 0.001 vs Control group; ^#^P < 0.05, ^##^P < 0.01, ^###^P < 0.001 vs Model group

Compared to the control group, treatment with LPS (1 μg/mL) and MSU (200 μg/mL) significantly increased the levels of IL-1β, IL-6, TNF-α, PGE2, and NO in the cell supernatant (P < 0.01). Compared to the model group, the levels of IL-1β, IL-6, TNF-α, PGE2, and NO were significantly decreased upon activation of Sirt1 (P < 0.05) but increased after inhibition of Sirt1 (P < 0.05) (Fig. 4). These results suggested the anti-inflammatory effect of Sirt1 activation and the pro-inflammatory effect of Sirt1 inhibition on gouty arthritis.

### Effect of activating and inhibiting Sirt1 on the levels of COX-2, iNOS and MCP-1 in THP-1 cells

**Fig. 5:**
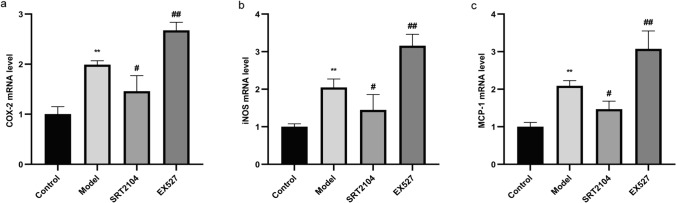
Effects of activating and inhibiting Sirt1 on the levels of COX-2 (**a**), iNOS (**b**), and MCP-1 (**c**) in LPS plus MSU-activated THP-1 cells. Data were shown as mean ± SD (n = 3); *P < 0.05, **P < 0.01, ***P < 0.001 vs Control group; ^#^P < 0.05, ^##^P < 0.01, ^###^P < 0.001 vs Model group

To further investigate the effects of inhibiting and activating Sirt1 on inflammatory mediators and chemokines, RT-qPCR was conducted. Compared to the control group, the expression of COX-2, iNOS, and MCP-1 was upregulated in the model group (all P < 0.01), decreased after Sirt1 activation, and increased again after Sirt1 inhibition (Fig. 5). These results confirmed that activation or inhibition of Sirt1 was associated with the release of inflammatory mediators and chemokines.

### Flow cytometry

#### Effects of activating or inhibiting Sirt1 on M1 and M2 phenotypes of macrophages.

**Fig. 6:**
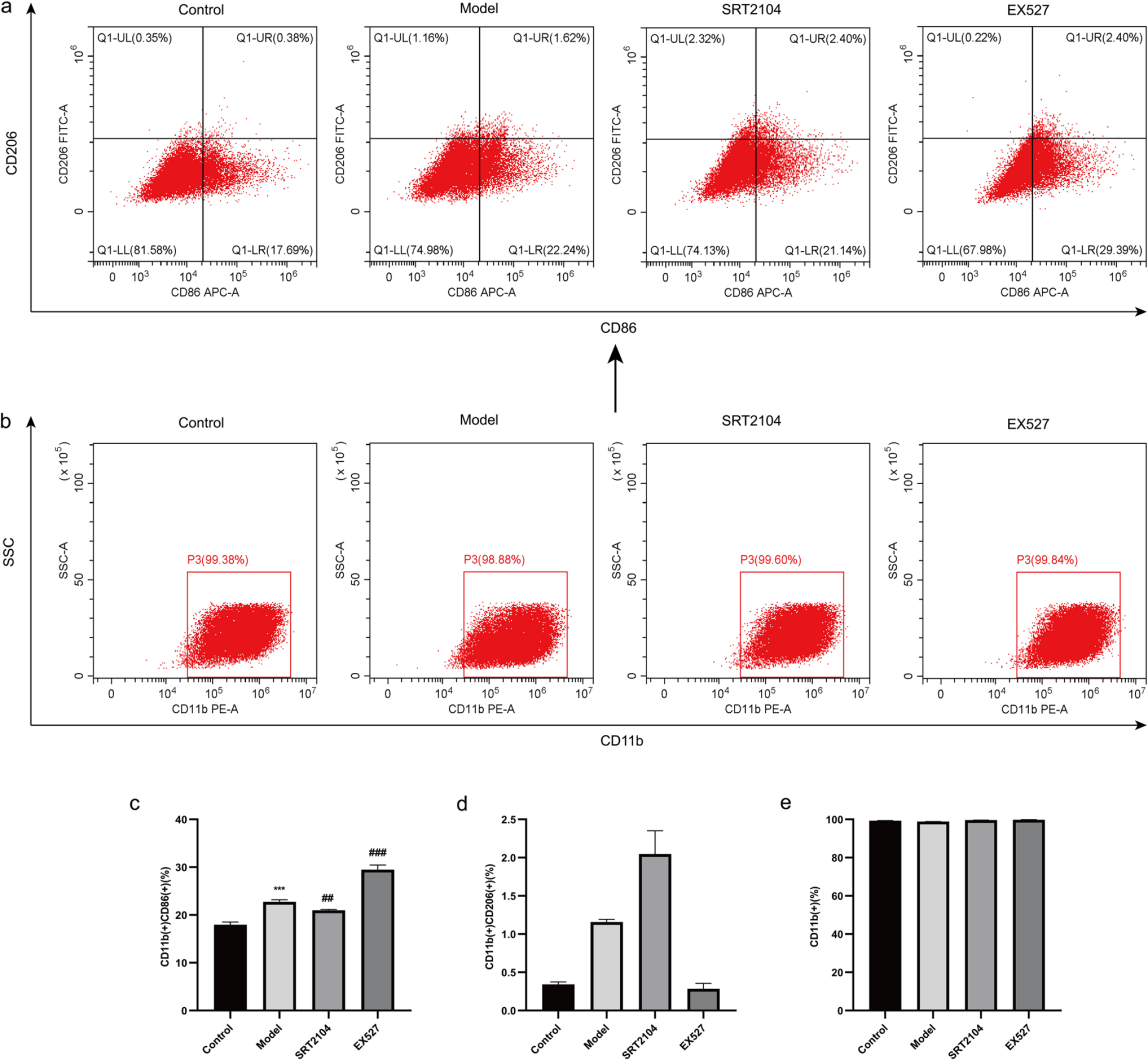
Effect of SRT2104 and EX527 on macrophage polarization. **a** Representative images of the proportions of CD11b(+) CD86(+) and CD11b(+) CD206(+) cells. **b** Representative images of the proportion of CD11b(+) cells. Statistical results of the proportions of CD11b(+) CD86(+) (**c**), CD11b(+) CD206(+) (**d**) and CD11b( +) (**e**) cells. Data were presented as mean ± SD (n = 3); *P < 0.05, **P < 0.01, ***P < 0.001 vs. Control group; ^#^P < 0.05, ^##^P < 0.01, ^###^P < 0.001 vs. Model group

To explore whether Sirt1 affects macrophage polarization, we intervened in Sirt1 expression in THP-1 cells and detected the M0 marker CD11b, the M1 marker CD86, and the M2 marker CD206. Compared to the control group, the proportion of CD11b(+) CD86(+) cells in the model group was significantly increased. Compared to the model group, activation of Sirt1 decreased the proportion of CD11b(+) CD86(+) cells, while inhibition of Sirt1 increased the proportion of CD11b(+) CD86(+) cells (Fig. 6a, c). Activation or inhibition of Sirt1 resulted in a lower proportion of CD11b(+) CD206(+) cells (Fig. 6a, d). Besides, the proportion of CD11b(+) cells was high, with no significant differences among the groups (Fig. 6b, e). Altogether, these results indicated that Sirt1 was associated with changes in M1 macrophage polarization status.

#### ROS

**Fig. 7:**
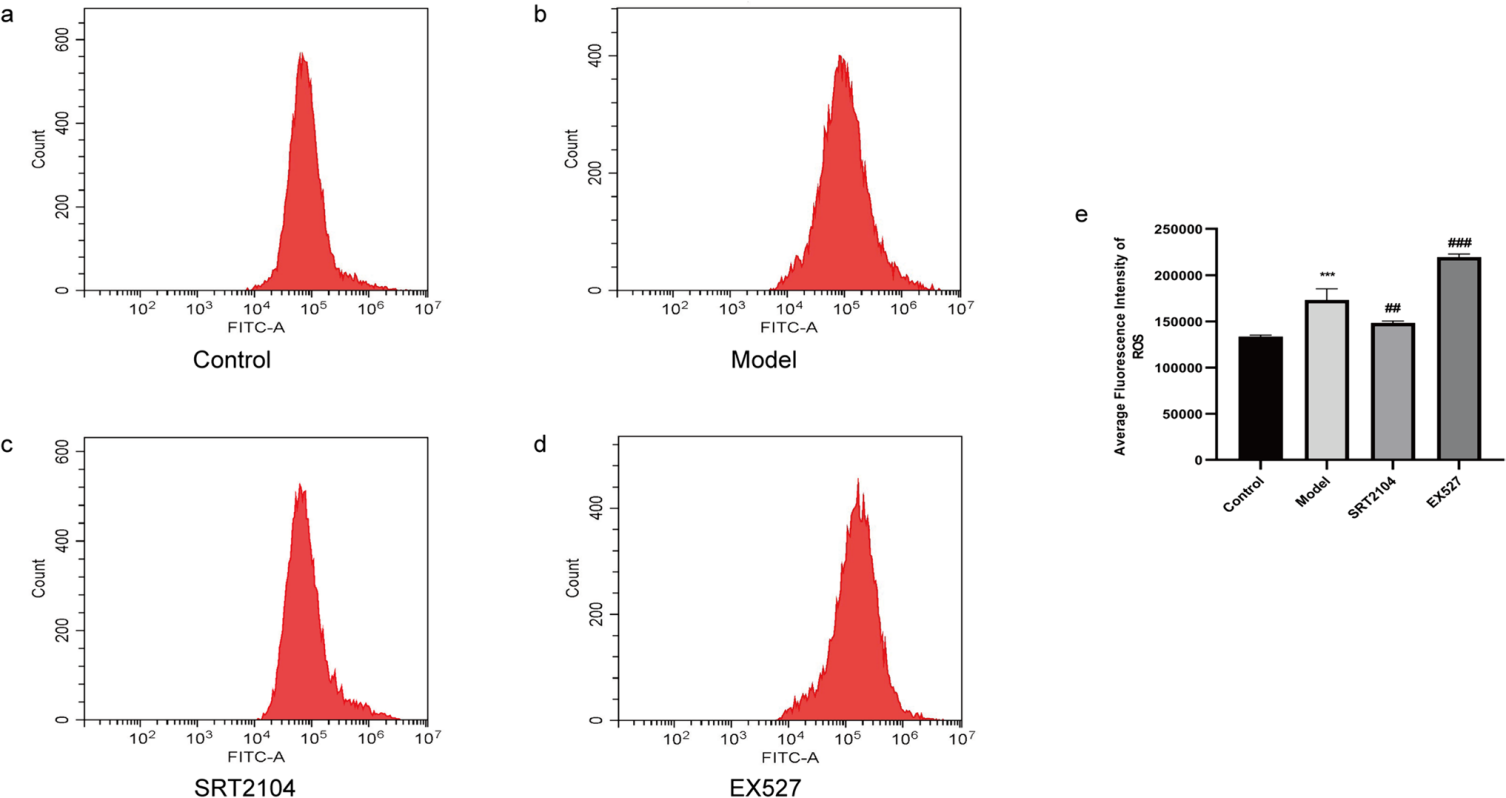
Effects of SRT2104 and EX527 on ROS production in LPS + MSU-activated THP-1 cells. **a–d** Representative images of fluorescence intensity values of ROS production in each group. **e** Statistical results of the average fluorescence intensity values of ROS. Data were presented as mean ± SD (n = 3); *P < 0.05, **P < 0.01, ***P < 0.001 vs Control group; ^#^P < 0.05, ^##^P < 0.01, ^###^P < 0.001 vs Model group

Compared to the control group, ROS production was enhanced in the model group. In comparison to the model group, ROS production declined after Sirt1 activation and increased after Sirt1 inhibition (Fig. 7). Overall, these results suggested that ROS production was associated with inflammation.

### Effects of activating or inhibiting Sirt1 on the MAPK/NF-κB/AP-1 and Nrf2/HO-1 signaling pathways in THP-1 cells

**Fig. 8:**
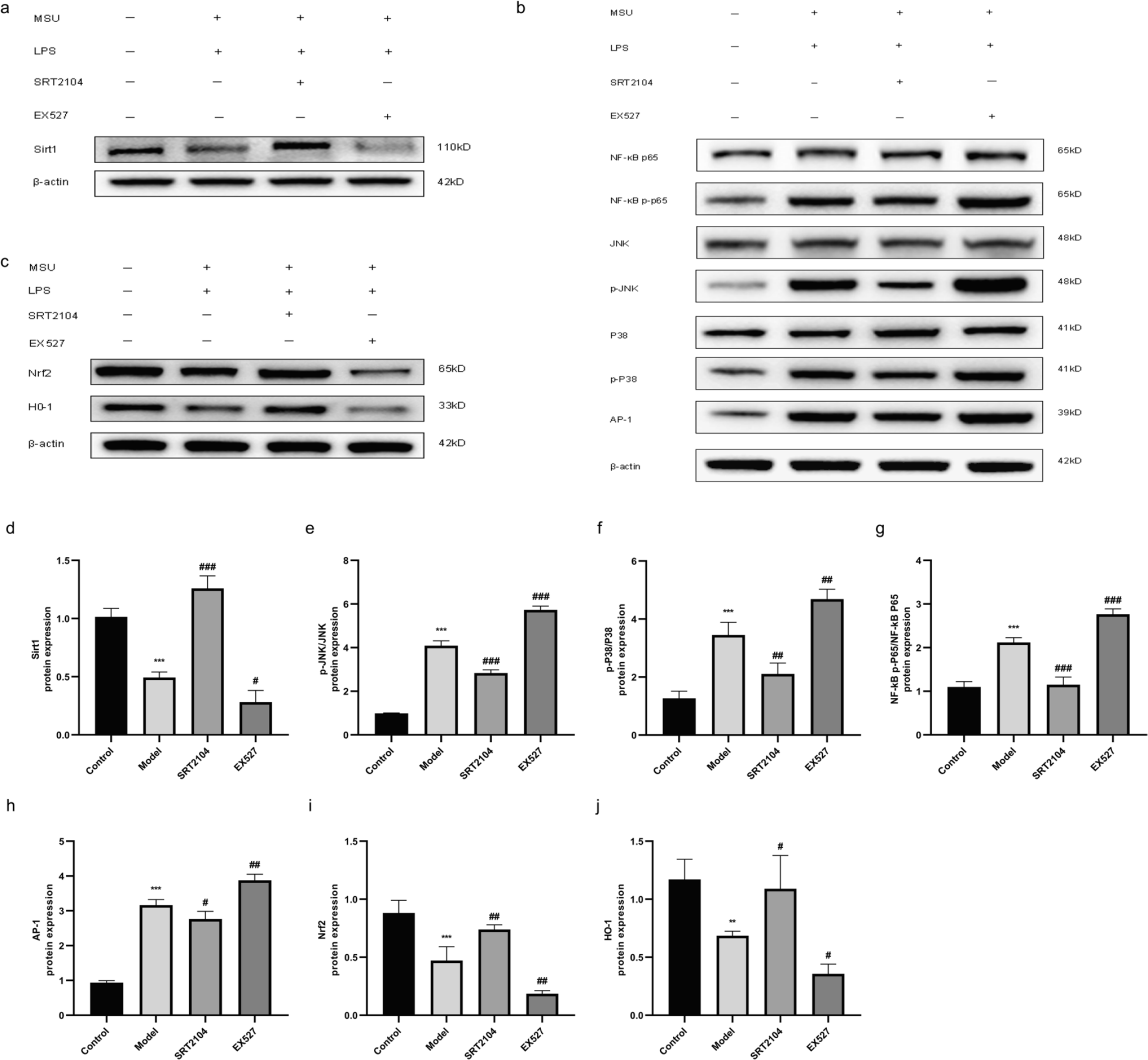
Effects of SRT2104 and EX527 on the MAPK/NF-κB/AP-1 and Nrf2/HO-1 signaling pathways in LPS + MSU-activated THP-1 cells. **a** Representative Western blot images of Sirt1 and β-actin. **b** Representative Western blot images of NF-κB p65, NF-κB p-p65, JNK, p-JNK, P38, p-P38, AP-1, and β-actin. **c** Representative Western blot images of Nrf2, HO-1, and β-actin. Changes in the relative protein levels of Sirt1 (**d**), p-JNK/JNK (**e**), p-P38/P38 (**f**), NF-κB p-p65/NF-κB p65 (**g**), AP-1 (**h**), Nrf2 (**i**), and HO-1 (**j**). Data were shown as mean ± SD (n = 3); *P < 0.05, **P < 0.01, ***P < 0.001 vs Control group; ^#^P < 0.05, ^##^P < 0.01, ^###^P < 0.001 vs Model group

Sirt1 protein levels in each group of THP-1 cells were detected after Sirt1 intervention. Sirt1 expression decreased in the model group, increased after Sirt1 activation (SRT2104), and further decreased after Sirt1 inhibition (EX527) (Fig. 8a, d).

To activate the MAPK signaling pathway, THP-1 cells were stimulated with LPS + MSU to release various pro-inflammatory cytokines. To further explore whether the anti-inflammatory effect of Sirt1 was related to the MAPK signaling pathway, we examined the expression of key proteins. It was found that the levels of p-JNK, p-P38, NF-κB p-p65, and AP-1 were significantly elevated in the model group (all P < 0.001), suppressed after activation of Sirt1, and enhanced after inhibition of Sirt1 (Fig. 8b, e–h). These results suggested that the association between the anti-inflammatory effect of Sirt1and the MAPK/NF-κB/AP-1 signaling pathway.

To further investigate the protective mechanism of Sirt1 against oxidative stress induced by LPS + MSU, we examined the expression of Nrf2 and HO-1 proteins. The expression of Nrf2 and HO-1 proteins was significantly downregulated in the model group (all P < 0.05), upregulated upon activation of Sirt1, and reduced again after inhibition of Sirt1(Fig. 8c, i–j). Collectively, these results identified that the protective effect of Sirt1 against oxidative damage was associated with the activation of the Nrf2/HO-1 signaling pathway.

## Discussion

This study highlighted that Sirt1 alleviated M1 macrophage polarization by inhibiting the MAPK/NF-κB/AP-1 signaling pathways and activating the Nrf2/HO-1 pathway, thereby attenuating the inflammatory response in gouty arthritis. In vivo*,* immunohistochemistry confirmed a significant increase in CD68(+) mononuclear macrophages and Sirt1 expression in the synovium during the recovery phase of gouty arthritis. In vitro, ELISA, NO assay, and RT-qPCR evinced that Sirt1 inhibited the release of inflammatory factors such as NO, PGE2, TNF-α, IL-1β, IL-6, COX-2, iNOS, and MCP-1. Flow cytometry and Western blot assays further proved that Sirt1 attenuated M1 polarization by suppressing the MAPK/NF-κB/AP-1 signaling pathway and activating the Nrf2/HO-1 pathway. In summary, our findings indicated that Sirt1 mitigated macrophage polarization and inflammatory responses in gouty arthritis through the MAPK and Nrf2/HO-1 pathways.

Gouty arthritis is characterized by sterile inflammation in the joints induced by MSU crystals. The crystal structures activate innate immune cells, especially macrophages, which are essential in the inflammatory response of gouty arthritis [23]. Throughout different stages of the inflammatory response, macrophages polarized to M1 or M2 phenotypes. In the early stages of gouty arthritis, macrophages tend to polarize towards the M1 phenotype, with more M1 macrophages as inflammation progresses, while in the later stages, they tend to polarize towards the M2 phenotype [24]. Sirt1, a class III deacetylase, has been shown to regulate inflammation and oxidative stress. Our study, through H&E staining and immunohistochemistry confirmed abundant macrophages and elevated Sirt1 expression in the synovium of a mouse model during the active phase of gouty arthritis. Additionally, the relationship between abundant macrophages and elevated Sirt1 levels was explored through in vitro experiments.

The regulatory effect of inflammation in gouty arthritis is closely associated with macrophage polarization. We attempted to explore whether intervention of Sirt1 levels in THP-1 macrophages could alter macrophage polarization and inflammation. Liu et al. [19] investigated the potential mechanisms of Sirt1 in acute gout and found that Sirt1 expression in peripheral blood mononuclear cells and synovium of patients with acute gout was much higher than that in patients with intercritical gout. Further studies revealed that downregulation of Sirt1 levels could promote M1 macrophage polarization. Our study also reached consistent conclusions. Flow cytometry results revealed that Sirt1 inhibition led to macrophage polarization towards the M1 phenotype, while Sirt1 activation inhibited macrophage polarization towards the M1 phenotype.

The MAPK signaling pathway and oxidative stress response are pivotal in stimulating inflammatory reactions. To further explore whether Sirt1 regulated macrophage polarization through the MAPK signaling pathway and oxidative stress response, we detected the levels of key proteins in the MAPK signaling cascade (P38, JNK, NF-κB p65, AP-1) and antioxidant stress response (Nrf2 and HO-1) by activating or inhibiting Sirt1 levels in THP-1 cells. Zhao et al. [25] conducted a study on the effect of Patatin-like phospholipase domain-containing protein 7 (PNPLA7) on macrophage polarization. The study found that overexpression of PNPLA7 increased Sirt1 levels and repressed p-P38 MAPK levels, thereby suppressing proinflammatory characteristics during LPS-induced M1 polarization, while knockdown of PNPLA7 yielded the opposite results. During the inflammatory response, macrophages produce substantial amounts of ROS, leading to oxidative stress and cellular and tissue damage. However, the Nrf-2/HO-1/NQO-1 signaling pathway enhances antioxidant and anti-inflammatory mechanisms to scavenge ROS and maintain intracellular redox balance [26]. A previous study found that Isoliquiritigenin (ISL) directly bound to Sirt1 and reduced inflammation and oxidative stress in vitro and in vivo through the MAPK and Nrf-2 signaling pathways [27]. However, there is no research available on the role of Sirt1 in alleviating macrophage polarization and inflammatory responses in gouty arthritis through the MAPK/NF-κB/AP-1 and Nrf2/HO-1 cascades. Our results first indicated that activation of Sirt1 increased Sirt1 expression in THP-1 cells and inhibited phosphorylation of P38 and/or JNK on the MAPK signaling pathway, as well as downstream phosphorylation of NF-κB p65 and activation of AP-1, thereby alleviating the inflammatory response. Furthermore, activation of Sirt1 reduced ROS generation, increased expression of Nrf-2 and HO-1, and activated antioxidant response signaling pathways, thereby alleviating the inflammatory response.

In summary, our study first demonstrates the crucial role and mechanisms of Sirt1 in regulating macrophage polarization and the inflammatory response in gouty arthritis through the MAPK and Nrf2/HO-1 pathways.

## Conclusion

Our study illuminates that Sirt1 mitigates macrophage polarization and inflammation in gouty arthritis by inhibiting the MAPK/NF-κB/AP-1 pathway and activating the Nrf2/HO-1 pathway, providing a new target for the treatment of gouty arthritis.

## Data Availability

All data generated or analyzed during this study are included in this published article (and its Supplementary Information files).
